# Number of initial symptoms of SARS-CoV-2 infection is associated with the risk of otological symptoms: a retrospective study

**DOI:** 10.1186/s12879-023-08866-w

**Published:** 2023-12-07

**Authors:** Qiang Wang, Hailing Gu, Yong Tao, Yu Zhao, Zhaoli Meng

**Affiliations:** 1 Department of Otolaryngology-Head and Neck Surgery, West China Hospital, Sichuan University, 37 Guo Xue Lane, Chengdu, 610041 Sichuan People’s Republic of China; 2 Department of Audiology and Speech Language Pathology, West China Hospital, Sichuan University, Chengdu, China

**Keywords:** SARS-CoV-2, Otological Diseases, Prevalence, Vaccine

## Abstract

**Background:**

The characteristics of otological symptoms in patients with severe acute respiratory syndrome coronavirus 2 (SARS-CoV-2) infection are lacking. Almost no research has been conducted to explore the emergence of otological symptoms after coronavirus disease 2019 infection. The aims of this study were to investigate the prevalence and specific clinical characteristics of and risk factors for otological symptoms among patients with SARS-CoV-2 infection.

**Methods:**

We included two groups to investigate the prevalence and clinical characteristics of otological symptoms among patients with SARS-CoV-2 infection. The first sample (S1) was drawn retrospectively from four communities via questionnaires, and the second sample (S2) from an outpatient clinic.

**Results:**

A total of 189 participants were included in S1 (124 women [65.6%]; mean [standard deviation (SD)] age, 33.66 [13.56] years), and 47 in S2 (25 women [53.2%]; mean [SD] age, 45.28 [14.64] years). The most prevalent otological symptoms in S1 were dizziness (15.9%), tinnitus (7.9%), aural fullness (6.9%), otalgia (5.3%), hearing loss (1.6%), and otopyorrhoea (1.1%). Moreover, for each additional typical symptom of SARS-CoV-2 infection, the risk (odds ratio) of otological symptoms increased by 1.33 (95% confidence interval: 1.10–1.61, *p* = 0.003). The prevalence of aural fullness was higher in the unvaccinated group than that in the group receiving two or three vaccinations (*p* = 0.018).

**Conclusions:**

Various otological symptoms may occur in patients with SARS-CoV-2 infection. The number of typical symptoms of SARS-CoV-2 infection is positively associated with the probability of otological symptoms. However, vaccination may reduce the probability of certain otological symptoms.

**Supplementary Information:**

The online version contains supplementary material available at 10.1186/s12879-023-08866-w.

## Background

In January 2020, the World Health Organization declared severe acute respiratory syndrome coronavirus 2 (SARS-CoV-2) as an international public health emergency, and, eventually, coronavirus disease 2019 (COVID-19) was declared a pandemic that affected people of all ages [1]. With the change in the Chinese government’s epidemic prevention policy in early December 2022, the number of SARS-CoV-2 infections in China increased significantly. According to data disclosed by the Chinese Center for Disease Control and Prevention, infection rates peaked at the end of December [2]. SARS-CoV-2 infection can be diagnosed using RNA, antigen, or antibody detection, or diagnostic imaging methods [3]. Rapid antigen self-testing is also effective for diagnosis, with a specificity of 97.5–99.5% [4]. Moreover, by combining symptoms with other information, such as contact or travel history, age, sex, and the recent local case detection rate, the sensitivity of disease prediction can reach 90% [5]. Patient self-reports of symptoms may be crucial in the identification of COVID-19 cases [6]. Patients with SARS-CoV-2 infection can be either asymptomatic or experience various symptoms, including pneumonia, fever, cough, sore throat, and smell and taste disorders [7]. As the virus continues to evolve, the symptomatic areas have been migrating from the lungs and nervous tissue to the upper respiratory tract [7]. An increasing number of studies are focused on the effects of SARS-CoV-2 infection in otolaryngology. The most common otorhinolaryngological symptoms of SARS-CoV-2 infection are smell and taste disorders, cough, sore throat, and dyspnoea [8].

SARS-CoV-2 may cause hearing loss, tinnitus, and dizziness. However, owing to insufficient evidence, such results should be carefully interpreted [9]. Certain studies indicate that SARS-CoV-2 may damage the audio-vestibular system, thereby resulting in sensorineural hearing loss (SNHL) [10–12], some cases of which may be severe [13]. SNHL may even be the only symptom of SARS-CoV-2 infection in certain cases [14]. Moreover, the incidence of vertigo and Meniere’s disease appeared to be raised during the COVID-19 pandemic [15]. However, other studies have suggested that new-onset hearing loss is not more common in patients with a positive SARS-CoV-2 test result than that in those with a negative result [16]. In one survey, 30% of patients infected with SARS-CoV-2 presented with tinnitus [17]. Some researchers have suggested that acute SARS-CoV-2 infection should be considered in patients experiencing dizziness [18]. In one case, a 37-year-old man infected with SARS-CoV-2 first presented with vertigo, fever, and diarrhoea [19]. However, the clinical significance of tinnitus and vestibular disturbances as symptoms of COVID-19 is unclear owing to their low incidences [16].

Almost no research has been conducted to explore the emergence of otological symptoms after COVID-19 infection. Therefore, the main aims of this study were to investigate the prevalence and specific clinical characteristics of and risk factors for various otological symptoms among patients with SARS-CoV-2 infection, which may shed further light on the effects of SARS-CoV-2 on humans and on symptom prevention.

## Methods

### Aims

To investigate the prevalence and specific clinical characteristics of and risk factors for various otological symptoms among patients with SARS-CoV-2 infection.

### Study participants

During the initial months after SARS-CoV-2 outbreak in Chengdu on 15 December 2022, an increasing number of patients visited the ear, nose, and throat outpatient clinic because of otological symptoms. To investigate and evaluate these symptoms, the first sample (S1) was drawn retrospectively from a community questionnaire and the second sample (S2) was drawn from the outpatient clinic. Patients aged > 18 years who were diagnosed with a SARS-CoV-2 infection were eligible for inclusion. According to the “Diagnosis and Treatment Protocol for Novel Coronavirus Infection-Induced Pneumonia” version 10 [20], we accepted three diagnostic methods for SARS-CoV-2 infection: positive results for a SARS-CoV-2 nucleic acid test [21] or rapid antigen self-test [22] and self-reported [23] typical symptoms during the initial months after the outbreak in Chengdu. If a patient was diagnosed based on more than one of these methods, priority was assigned in the following order: nucleic acid test > rapid antigen self-test > self-reported symptoms. Ethical approval was obtained from the West China Hospital of Sichuan University Biomedical Research Ethics Committee (no. 2023 (137)). Participants were enrolled after they provided written informed consent.

### Questionnaires (S1)

A questionnaire-based survey was randomly conducted in four communities in Chengdu in January 2023 to eliminate survivorship bias. Participants were asked to complete a questionnaire that we developed considering the various symptoms they experienced during the SARS-CoV-2 outbreak, in retrospect. This questionnaire was designed according to the purpose of this study. To ensure the validity of the questionnaire, it was validated by five otorhinolaryngologists, an epidemiologist and a statistician. We started the survey about 1 month after the start of the COVID-19 pandemic in the area. The baseline questionnaire included demographics, diagnostic criteria, vaccine dose, and information on initial symptoms (including typical SARS-CoV-2 symptoms: fever, cough, fatigue, headache, nasal obstruction, muscular pain, pharyngalgia, smell and taste disorders, and other problems [e.g., chest pain and insomnia]) and otological symptoms (e.g., tinnitus, otalgia, hearing loss, aural fullness, otopyorrhoea, and dizziness). Furthermore, data were collected regarding the occurrence and duration of initial and otological symptoms, number of days between infection diagnosis and symptoms, recurrence, aggravation of otological symptoms, disappearance of symptoms, days between onset and disappearance of symptoms, and whether treatment was sought. An additional text file provides further details regarding the questionnaire. [see Additional file 1]

### Outpatient clinics (S2)

In order to explore the specific manifestations of otological symptoms after infection with COVID-19, such as the patient’s hearing thresholds andthe type of hearing loss, we invited people with otological symptoms after COVID-19 infection online for further specialist examination at the same time. None of them were participants in S1. This group of patients did not have any otological symptoms before infection with COVID-19. Medical history-taking and audiometric assessments were performed as a part of routine medical treatment at the outpatient clinic. These included the assessment of tinnitus laterality as well as bilateral otoscopy, tympanometry, and pure-tone audiometry. The pure-tone average (PTA) threshold was calculated for each ear at four frequencies: 500, 1000, 2000, and 4000 Hz. PTA thresholds in the better ear (BEPTAs) were used to define hearing categories according to the guidelines of the World Health Organization (1997) [23]. The speech recognition rate, also known as the word recognition score, was recorded using speech audiometry.

### Statistical analysis

All continuous variables are presented as means and standard deviations (SDs), and categorical data are presented as numbers (percentages). Chi-squared test or Fisher’s exact test was used to compare categorical variables among groups, and Student’s t-test or one-way analysis of variance was used to compare continuous variables among groups, as appropriate. A logistic regression model was used with the assumption that the categorical outcomes of otological symptoms changed with age, sex, vaccine dose, and the number of typical SARS-CoV-2 symptoms. The prevalence of otological symptoms was calculated for three groups of patients according to the number of vaccine doses.

Data analyses were performed using an open-source statistical analysis software (R version 4.0.5; The R Foundation for Statistical Computing, Vienna, Austria) and the GraphPad Prism 8.3.0 software (GraphPad Software, San Diego, California, USA). All statistical tests were two-sided; *p* values below 0.05 were regarded significant.

## Results

### Baseline characteristics and prevalence of otological symptoms

Of the 200 participants invited to participate in the questionnaire, a cohort of 189 with complete data was included in S1 (124 women [65.6%] and 65 men [34.4%]; mean [SD] age, 33.7 [13.6 years). Of all the participants, 48.7%, 39.7%, and only 11.6% were diagnosed with SARS-CoV-2 infection via self-reported symptoms, rapid antigen self-testing, and SARS-CoV-2 nucleic acid testing, respectively. In addition, 92.1% of the patients received at least one dose of the available SARS-CoV-2 vaccines. The baseline characteristics of S1 according to otological symptoms are summarised in Table 1.

**Table 1:** Baseline characteristics of S1, stratified according to otological symptoms

Characteristics	Overall	With	Without	*P* value
	(N = 189)	(N = 56)	(N = 133)	
Sex (%)				1.000
F	124 (65.6)	37 ( 66.1)	87 ( 65.4)	
M	65 (34.4)	19 ( 33.9)	46 ( 34.6)	
Age (years, mean (SD))	33.66 (13.56)	33.11 (13.04)	33.89 (13.81)	0.719
Diagnostic criteria (%)				0.069
Positive SARS-CoV-2 nucleic acid	22 (11.6)	8 ( 14.3)	14 ( 10.5)	
Rapid antigen self-test	75 (39.7)	28 ( 50.0)	47 ( 35.3)	
Self-reported	92 (48.7)	20 ( 35.7)	72 ( 54.1)	
Vaccine dose (%)				0.250
0	15 ( 7.9)	2 ( 3.6)	13 ( 9.8)	
1	3 ( 1.6)	2 ( 3.6)	1 ( 0.8)	
2	26 (13.8)	7 ( 12.5)	19 ( 14.3)	
3	145 (76.7)	45 ( 80.4)	100 ( 75.2)	
Initial symptoms duration (days, mean (SD))	5.56 (4.23)	5.24 (3.43)	5.69 (4.53)	0.505

The main prevalence rates of otological symptoms, demographic characteristics, symptom duration, and recurrence of patients with otological symptoms in S1 are summarised in Table 2. Overall, 56 participants reported having had SARS-CoV-2-associated otological symptoms, as follows: dizziness (15.9%), tinnitus (7.9%), aural fullness (6.9%), otalgia (5.3%), hearing loss (1.6%), and otopyorrhoea (1.1%). The interval between diagnosis and otological symptoms after was similar among the categories (dizziness: 3.1 days, tinnitus: 4.0 days, aural fullness: 2.9 days, otalgia: 3.3 days, hearing loss: 4.0 days, and otopyorrhoea: 5 days). The mean (SD) duration of otological symptoms was 7.31 (7.41) days. More than half of the participants reported that their otological symptoms persisted at the time of completing the questionnaire, but only two of them sought medical treatment.

**Table 2:** Clinical characteristics of patients with otological symptoms in S1 (N = 56)

Characteristics	Aural Fullness	Dizziness	Hearing Loss	Otalgia	Otopyorrhea	Tinnitus	*P* value
	(N = 13)	(N = 30)	(N = 3)	(N = 10)	(N = 2)	(N = 15)	
Prevalence	6.9%	15.9%	1.6%	5.3%	1.1%	7.9%	
Sex (%)							0.735
F	9 (69.2)	23 ( 76.7)	3 (100.0)	6 (60.0)	1 ( 50.0)	11 (73.3)	
M	4 (30.8)	7 ( 23.3)	0 ( 0.0)	4 (40.0)	1 ( 50.0)	4 (26.7)	
Age (years, mean (SD))	32.00 (13.21)	32.10 (11.21)	64.00 (23.07)	26.50 (7.53)	38.00 (4.24)	32.53 (12.68)	**0.001**
Vaccine Dose (mean (SD))	3.00 (0.00)	2.70 (0.60)	2.00 (1.73)	2.90 (0.32)	3.00 (0.00)	2.67 (0.82)	0.193
Interval time (days, mean (SD))	2.92 (1.80)	3.10 (4.33)	4.00 (1.00)	3.30 (1.25)	5.00 (2.83)	4.00 (4.07)	0.924
Recurrence (%)							0.778
Initial	6 (46.2)	14 ( 46.7)	2 ( 66.7)	5 (50.0)	0 ( 0.0)	8 (53.3)	
Recurrence	7 (53.8)	16 ( 53.3)	1 ( 33.3)	5 (50.0)	2 (100.0)	7 (46.7)	
Symptoms worsen (%)							**0.015**
No	9 (69.2)	30 (100.0)	2 ( 66.7)	7 (70.0)	1 ( 50.0)	14 (93.3)	
Yes	4 (30.8)	0 ( 0.0)	1 ( 33.3)	3 (30.0)	1 ( 50.0)	1 ( 6.7)	
Otological symptoms disappeared (%)							0.683
No	5 (38.5)	16 ( 53.3)	2 ( 66.7)	5 (50.0)	2 (100.0)	8 (53.3)	
Yes	8 (61.5)	14 ( 46.7)	1 ( 33.3)	5 (50.0)	0 ( 0.0)	7 (46.7)	

Similarly, for S2, a total of 47 patients were willing to undergo specialist examinations. The medical history of these 47 patients was collected at the outpatient clinic. Their demographic data, diagnostic criteria, and vaccine doses are summarised in Table 3. These participants retrospectively reported having the following SARS-CoV-2-associated otological symptoms: tinnitus (61.7%), hearing loss (55.3%), aural fullness (29.8%), otalgia (19.1%), otopyorrhoea (4.3%), and dizziness (4.3%). In addition, tympanograms were normal for 58 ears and abnormal for 36. Tympanograms were type B, type C, and irregular for 2, 12, and 23 ears, respectively. The mean (SD) BEPTA of S2 was 22.26 (17.23) dB HL. Of the 47 participants, 22.3% had SNHL, and 24.5% had mixed hearing loss.

**Table 3:** Baseline characteristics of S2 (N = 47)

Characteristics		Overall		Characteristics	Overall
Sex (%)	F	25 (53.2)		**Otological Symptoms**	
	M	22 (46.8)		Aural Fullness (%)	14 (29.8)
Age (mean (SD))		45.28 (14.64)		Dizziness (%)	2 ( 4.3)
Diagnostic criteria (%)	Positive SARS-cov-2 nucleic acid	7 (14.9)		Hearing Loss (%)	26 (55.3)
	Rapid antigen self-test	26 (55.3)		Otalgia (%)	9 (19.1)
	Self-reported	14 (29.8)		Otopyorrhea (%)	2 ( 4.3)
Vaccine dose (mean (SD))		2.53 (0.88)		Tinnitus (%)	29 (61.7)

**Fig. 1:**
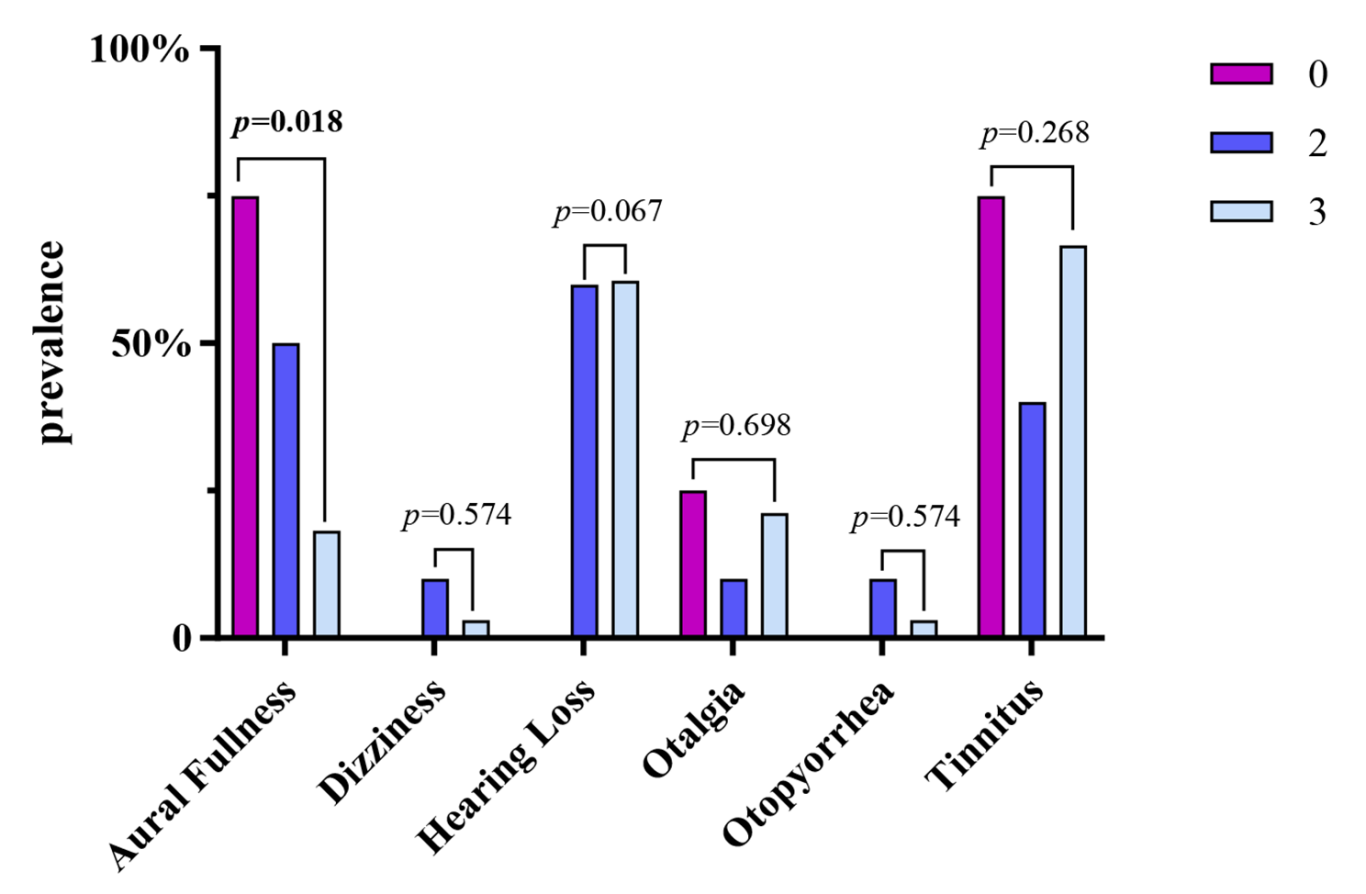
Prevalence of otological symptoms in different vaccine dose groups among patients with SARS-CoV-2 infection. 0: group without vaccination; 2: group with two doses of vaccination; 3: group with three doses of vaccination

The prevalence of aural fullness was higher in the unvaccinated group than that in the group that received two or three vaccinations (*p* = 0.018, Fig. 1). In S2, 2 patients had initial dizziness and 26 had hearing loss after SARS-CoV-2 infection. Patients with SARS-CoV-2 infection with or without hearing loss differed in terms of the number of vaccination doses received (*p* = 0.038), and the mean (SD) interval between the diagnosis and onset of otological symptoms among patients with hearing loss in S2 was 5.31 (2.98) days. Nine participants in S2 presented with initial otalgia, at an interval of 5.00 (3.81) days, and women were more likely to experience otalgia than men (*p* = 0.044). Only two participants had otopyorrhoea, with abnormal tympanograms and pure-tone audiograms both revealing conductive hearing loss. Tinnitus was the most common otological symptom in S2, reported by 29 participants, of which 4 had previously had tinnitus. Among them, the interval between diagnosis and onset of tinnitus was 5.45 (3.53) days. In 5, 15, and 9 patients the tinnitus was left-sided (17.2%), right-sided (51.7%), and bilateral (31.0%), respectively. Interestingly, the mean BEPTA in the group without tinnitus was lower than that in the group with tinnitus (*p* = 0.023).

### Risk factors among patients with otological symptoms after SARS-CoV-2

Multiple logistic regression revealed that the number of typical symptoms of SARS-CoV-2 was an independent risk factor for otological symptoms (odds ratio, 1.33; 95% confidence interval, 1.10–1.61, *p* = 0.003). Thus, for each additional typical symptom of SARS-CoV-2, the risk of developing otological symptoms increased 1.33 times. However, age, sex, and the number of vaccination doses were not associated with otological symptoms (all *p* > 0.05).

## Discussion

The COVID-19 pandemic has posed a considerable threat to human health. As SARS-CoV-2 continues to evolve, the range of symptoms of infection are increasing. In this study, two samples were used to identify the baseline characteristics of patients, prevalence of various otological symptoms, and risk factors among patients with otological symptoms that developed after COVID-19 diagnosis. Interestingly, we discovered that the prevalence of typical symptoms of SARS-CoV-2 was positively associated with the risk of otological symptoms. Moreover, the vaccine may protect against the risk of aural fullness and hearing loss in patients with SARS-CoV-2. These results contribute to the body of knowledge of the effects of SARS-CoV-2 infection on humans and may contribute to symptom prevention.

In our study, aural fullness, otalgia, and otopyorrhoea were common otological symptoms of SARS-CoV-2 infection. These are also the main symptoms of otitis media [24]. In previous studies, children were much more likely to develop otitis media than adults because of the anatomical characteristics of the eustachian tube [25]. However, adults who have been diagnosed with COVID-19 also experience the abovementioned three otological symptoms. Previous studies have demonstrated that a functional eustachian tube protects the middle ear from bacteria and viruses [26]. However, in ferrets, the influenza A virus can impair ventilation of the eustachian tube, leading to otitis media [27]. As SARS-CoV-2 continues to evolve, patients tend to develop upper respiratory symptoms, such as rhinorrhoea, nasal congestion, and cough. These symptoms are similar to those caused by the influenza A virus. Moreover, both SARS-CoV-2 and influenza viruses can cause inflammation of the upper respiratory mucosa [28]. Therefore, we infer that SARS-CoV-2 may damage the normal function of the eustachian tube, causing patients to experience otalgia, aural fullness, and otopyorrhoea.

In our study, hearing loss, tinnitus, and dizziness were also common otological symptoms in patients with SARS-CoV-2 infection. In S2, both conductive hearing loss and SNHL were observed. The presence of SNHL, tinnitus, and dizziness in patients infected with COVID-19 may reflect the neurophilic and neuroinvasive characteristics of SARS-CoV-2. SARS-CoV-2 can cause damage to the central and peripheral nervous systems [29]. Previous studies have suggested that viruses, including the measles, herpes, and mumps viruses, are important causes of damage to the audio-vestibular system, resulting in SNHL, tinnitus, and dizziness [30, 31]. Despite the lack of adequate pathological studies, previous reports [14, 32] and the present study suggest that SARS-CoV-2 poses a substantial risk of damaging the audio-vestibular system.

Furthermore, our research demonstrated a positive association between typical symptoms of SARS-CoV-2 infection and the probability of otological symptoms. However, according to our results, vaccines may reduce the risk of aural fullness and hearing loss in patients infected with SARS-CoV-2. A prospective cohort study revealed that the number of symptoms of SARS-CoV-2 infection is related to the risk of long COVID-19 [33]. Another study demonstrated that patients infected with SARS-CoV-2 who had received a SARS-CoV-2 vaccine cleared SARS-CoV-2 faster than those who did not receive the vaccine [34]. The SARS-CoV-2 vaccine effectively reduces the viral load of SARS-CoV-2, as well as the incidence and severity of SARS-CoV-2 symptoms [35, 36]. SARS-CoV-2 vaccination reduces the risk of successful invasion of the virus into the body because of its rapid elimination, which is consistent with our results.

This study has several limitations. First, the large number of infections has had a large impact on the medical system. Therefore, we had limited access to data. Second, because our survey relied on patients’ memories, it was subject to recall bias especially regarding the results of symptom durations. In addition, despite the fact that the accuracy of self-reporting COVID-19 can be up to 90% when combining patients’ self-reported symptoms, positive detection rate at residence, contact with positive patients and other factors [5], some of the patients we included were not identified as positive for infection via antigen or nucleic acid testing owing to lack of healthcare resources. This might have biased our results. In future, research similar to ours should be carried out in different locations and with larger samples, toward the prevention of symptoms following COVID-19. In addition, although most otological symptoms will self-heal, treatment methods for patients with long-lasting otological symptoms should be investigated.

## Conclusions

In this study, a variety of otological symptoms occurred in patients diagnosed with SARS-CoV-2 infection. The number of initial symptoms of SARS-CoV-2 infection was positively associated with the probability of otological symptoms. However, vaccination may reduce the risk of otological symptoms. We suggest that people at a high risk of hearing loss, including those with previous ear diseases, older people, and those who have not been vaccinated, should be alerted to the risk of otological symptoms following SARS-CoV-2 infection. Timely vaccination is important to prevent such otological symptoms.

### Electronic supplementary material

Below is the link to the electronic supplementary material.

**Additional file 1:** Questionnaire details

## Data Availability

The datasets used and/or analysed during the current study are available from the corresponding author on reasonable request.

## List of abbreviations

BEPTA: Better-ear pure-tone average
COVID-19: Coronavirus disease 2019
PTA: Pure-tone average
SARS-CoV-2: Severe acute respiratory syndrome coronavirus 2
SD: Standard deviation
SNHL: Sensorineural hearing loss
