# Supplementary material for: Number of initial symptoms of SARS-CoV-2 infection is associated with the risk of otological symptoms: a retrospective study
Source: BMC Infect Dis. 2023 Dec 7;23:862. doi: 10.1186/s12879-023-08866-w (PMC10704705; doi:10.1186/s12879-023-08866-w)
Supplement: Supplementary file 1 — Additional file 1: Questionnaire details [file 12879_2023_8866_MOESM1_ESM.docx]

Questionnaire details

1. Name
2. Gender
3. Age
4. Date of COVID-19 infection
5. Evidence of COVID-19 infection

- Self-reported symptoms
- Positive antigen test
- Positive nucleic acid test
- Other

1. Symptoms of COVID-19 infection

- Fever
- Cough
- Fatigue
- Headache
- Nasal obstruction
- Muscular pain
- Pharyngalgia
- Smell and taste disorders
- Other problems

1. Whether or not vaccinated

- Yes, vaccine dose
- No

1. Duration of COVID-19 infection initial symptoms (days)
2. Otological symptoms after COVID-19 infection

- Tinnitus
- Otalgia
- Hearing loss
- Aural fullness
- Otopyorrhoea
- Dizziness
- None

1. Duration of otological symptoms(days)
2. Number of days between infection diagnosis and symptoms
3. Did you experience any of the following symptoms prior to being infected with COVID-19?

- Tinnitus
- Otalgia
- Hearing loss
- Aural fullness
- Otopyorrhoea
- Dizziness
- None

1. Whether or not otological symptoms are a recurrence of past symptoms

- Yes
- No

1. Whether or not otological symptoms have disappeared

- Yes
- No
